# Calcifying algae maintain settlement cues to larval abalone following algal exposure to extreme ocean acidification

**DOI:** 10.1038/s41598-017-05502-x

**Published:** 2017-07-18

**Authors:** Jennifer K. O’Leary, James P. Barry, Paul W. Gabrielson, Laura Rogers-Bennett, Donald C. Potts, Stephen R. Palumbi, Fiorenza Micheli

**Affiliations:** 1 Hopkins Marine Station, Stanford University, Monterey, Pacific Grove, United States of America; 2 California Sea Grant, Department of Biology, California Polytechnic State University, San Luis Obispo, United States of America; 3 Monterey Bay Aquarium Research Institute, Moss Landing, California, United States of America; 4 Biology Department, Herbarium, University of North Carolina, Chapel Hill, North Carolina, United States of America; 5 Bodega Marine Laboratory, University of California, Davis, California, United States of America; 6 California Department of Fish and Wildlife, Marine Region, Bodega Bay, California, United States of America; 7 Department of Ecology and Evolutionary Biology, University of California, Santa Cruz, California, United States of America

## Abstract

Ocean acidification (OA) increasingly threatens marine systems, and is especially harmful to calcifying organisms. One important question is whether OA will alter species interactions. Crustose coralline algae (CCA) provide space and chemical cues for larval settlement. CCA have shown strongly negative responses to OA in previous studies, including disruption of settlement cues to corals. In California, CCA provide cues for seven species of harvested, threatened, and endangered abalone. We exposed four common CCA genera and a crustose calcifying red algae, *Peyssonnelia* (collectively CCRA) from California to three pCO_2_ levels ranging from 419–2,013 µatm for four months. We then evaluated abalone (*Haliotis rufescens*) settlement under ambient conditions among the CCRA and non-algal controls that had been previously exposed to the pCO_2_ treatments. Abalone settlement and metamorphosis increased from 11% in the absence of CCRA to 45–69% when CCRA were present, with minor variation among CCRA genera. Though all CCRA genera reduced growth during exposure to increased pCO_2_, abalone settlement was unaffected by prior CCRA exposure to increased pCO_2_. Thus, we find no impacts of OA exposure history on CCRA provision of settlement cues. Additionally, there appears to be functional redundancy in genera of CCRA providing cues to abalone, which may further buffer OA effects.

## Introduction

Predictions of marine ecosystem function under future ocean acidification (OA) suggest that the acidification rate^1–3^ is likely to overwhelm the capacity of many species to respond^4^ because OA is creating conditions organisms may not have experienced in their evolutionary history^5^. Further, the impacts of OA on single species or groups of species may cascade through ecosystems in ways that alter overall species diversity and ecosystem functions^6^, especially when the target species have critical ecological roles, such as habitat or food for other organisms. Because most ocean acidification studies focus on individual species, rather than species interactions^7^, understanding how OA alters interactions and ultimately ecosystem functioning remains a major knowledge gap^3, 6, 8^ and research priority^9, 10^.

The strongest potential impacts of OA are on marine calcifiers^1, 6^. Calcified structures occur widely across many phyla and have evolved independently and repeatedly over geologic time^4^. Calcifying organisms are abundant in marine systems and include many holoplankton, benthic invertebrates, and benthic algae. If calcifiers are unable to adapt to OA-induced seawater changes, there may be major changes throughout marine communities, because numerous non-calcifying organisms rely on calcifiers for food or biogenic habitat^1^. Crustose coralline algae (CCA) appear to be the calcifiers most vulnerable to OA^6^ because they deposit high-Mg calcite, the most soluble form of biogenic CaCO_3_^11^. In experiments with even relatively mild increased pCO_2_ (600–850 µatm), up to 100% reduction in CCA growth or cover have been reported^12–14^. CCA are not only widespread and abundant (forming 25–70% of the benthos in tropical and temperate reefs^15–17^), but also play important ecological roles including substrate consolidation, providing food for grazers, and providing space and cues for invertebrate larval settlement^15, 18, 19^. In addition to CCA, crustose and calcifying but non-coralline red algae from the genus *Peyssonnelia* are common on the world’s shelves^20^. Unlike CCA, *Peyssonnelia* deposit aragonitic calcite. The majority of studies on *Peyssonnelia* are from the tropics and Mediterranean, or from rhodolith beds, but *Peyssonnelia* has been found as a common substate in at least parts of the Pacific coast of the USA^21^.

The role CCA (and potentially other red algal crusts) play in providing cues for invertebrate larval settlement is critical: loss or change in coralline crusts can cause dramatic reduction in recruitment^15, 22^, thus indirectly altering invertebrate population dynamics. Diverse taxa (including sea urchins, abalones, limpets, scleractinian corals, and octocorals) have chemosensory systems that recognize particular chemical cues from CCA or their associated bacterial biofilms that induce larval settlement^19^. At least one type of non-coralline red algae induces abalone larval settlement (*Hildenbrandia dawsonii*)^23^.Thus, changes in the settlement cues provided by crustose calcifying red algae (CCRA, including both CCA and other calcifying red crusts) may have ecosystem-level consequences, mediated by changes in settlement of invertebrate species^19, 24^. Previous studies show that OA may reduce CCA coverage, thickness, skeletal strength, or physiological properties. The few studies that have focused on the effects of OA on CCA cues provided to settlers have focused on corals, and acidification of CCA led to reductions in coral settlement of up to 86% due to changes in settlement cues^25, 26^.

In the temperate rocky reefs of California, CCRA comprise ~30% of benthos (JKO unpublished data) and are critical settlement substrates for seven species of California abalone, including endangered species (the white and black abalone, *Haliotis sorenseni* and *H. cracherodii*) and the recreationally fished red abalone (*H. rufescens*). Despite several studies on the impacts of OA on larval development, growth, and survival of abalone in the California Current^27–29^ there have been no studies to date that evaluate the potential indirect impacts of OA on abalone larval settlement. During the upwelling season, over a period of several months CCRA are exposed to repeated low pH events, raising the question of how this exposure may affect their properties as a settlement substrate for invertebrate larvae. While abalone larvae may also be affected by exposure to OA, larvae are in their pelagic larval phase for only 5–10 days^30^ and may not see low pH conditions during this period. Exposure of tropical CCRA to OA has led to changes ﻿in both larval recruitment rate and larval preference for algal substrates^25^.

We grew four genera of common CCRA from California kelp forests under three pCO_2_ levels ranging from normal to extreme (419–2,013 µatm) to produce living algae differing only in pCO_2_ history. We used these algae in trials to determine whether abalone have different settlement rates on common CCRA, and whether algal pCO_2_ exposure history affects interactions between the algae and larval abalone settlers when only the algae are exposed to OA conditions. We hypothesized that elevated pCO_2_ would greatly diminish abalone settlement through changes in settlement cues, but with differential effects across algal genera. Thus, we also hypothesized that the importance of any OA effects on CCRA for abalone would depend on whether susceptible algae were also preferred by abalone settlers.

## Methods

### Algal collection and preparation

We collected algae crusts from cobbles at 9 m depth in kelp forests in the Monterey Bay, California, in February 2013. CCRA covered cobbles are the primary abalone settlement habitat^31^. Fifteen cobbles (1000 to 4000 cm^3^) were collected at Lovers Point (N36°37′ 32.65, W121°54′ 54.55) and 15 from the Hopkins Marine Reserve (HMR, N36°37′ 10.57, W121°54′ 11.61) and stored in running seawater at the HMR under ambient Monterey Bay temperature (~11.7 °C) and pH (~7.81) until early March 2013. Fluorescent bulbs provided light on a 12 hour cycle at ~15 µmol/m^2^/s, approximating average light levels measured at mid-day under kelp canopy at 9 m depth at HMR in October 2011 and March 2012 (LI-COR PAR measurement unit with an LI-193 spherical quantum sensor). Thin rock pieces (30–405 mm^2^) covered with a single algal morphology were cut from the cobbles using a rock saw, maintained in running seawater, and examined under an Olympus SZX-ILLD100 dissecting scope to ensure that no invertebrates were attached or embedded. Each piece was assigned to one of five morphologically distinct groups based on: type of reproductive structure (conceptacle versus nemathecium), size, and distribution; surface growth patterns; growth margins; crust thickness; and hypothallus cell arrangement (for CCA only).

To identify each morphological group, we sequenced 5–10 representatives per group. After extracting total genomic DNA for CCRA^32, 33^, we amplified partial sequences from two plastid encoded genes (rbcL and psbA) used extensively to distinguish genera and species of CCRA^32, 34^. Amplification and sequencing protocols were those of ^33^. Sequences were obtained from an ABI 3100 Genetic Analyzer at the University of North Carolina, Wilmington (see^32^). Identifications were based on sequencing ~20% of the *rbc*L gene and matching with sequences from curated type specimens. Reference samples of each sequenced crust have been deposited in the University of North Carolina Herbarium (NCU) herbarium. Four of the five morphological groups were taxonomically consistent to genus, and these were used in the experiments.

We created thirty replicate vials per algal taxon on March 2, 2013 by epoxying 1–3 pieces of alga into clear polystyrene vials (47.75 mm diameter ×102.12 mm height, 147 mL) using Z-spar epoxy compound. The living algal surfaces were flush with the epoxy, ~20 mm above the vial bottom, with space to grow laterally to at least double the initial area. Vials were open at the top and submerged in seawater in an upright position at the Hopkins Marine Station (conditions described above). Crust area was measured from digital photographs (Canon Powershot S100) using Image J (http://rsbweb.nih.gov/ij/). On March 7, 2013 the vials were transported in cooled seawater to the Monterey Bay Aquarium Research Institute (MBARI).

### Algal pCO_2_ Conditioning

Experimental water was prepared at three pCO_2_ levels: ambient, high and extreme (Table 1a). The ambient treatment (418.5 µatm pCO_2_; pH 9.2; Ω_arag_ 1.81) represented non-upwelling conditions in central California (pH 7.7–8.1)^35^. The high pCO_2_ treatment (1,175.5 µatm; pH 7.52; Ω_aragonite_ 0.77) resembled the IPCC 2007 high-level projections for global oceanic pCO_2_ (worst-case stabilization level VI, pCO_2_ > 900)^36^ and the worst-case representative carbon pathway (RCP 8.5)^37^. In the Monterey Bay, this “high” level of pCO_2_ can occur during upwelling events. The extreme treatment (2,012.9 µatm; pH 7.31; Ω_aragonite_ 0.49) exceeded both predictions for the year 2100^33^ and levels found in nearshore waters during strong upwelling^38, 39^. We report Ω_aragonite_ because the solubility of high-Mg calcite is closer to aragonite than calcite^24^ (see Table 1a for Ω_calcite_).

**Table 1:** (a) Water chemistry set points (mean ± 1 SE) in gas-control tanks at MBARI, measured continuously every 60 seconds between March 7 and July 10, 2013. (b) Water chemistry as measured in the bins containing coralline algae and calculations of pCO_2_ and Ω (from pH and total alkalinity). Salinity was 33.9 ± 0.05 ppm.

(a)						
Treatment	pH	O_2_ (µM)	Temperature (°C)			
Ambient	7.906 ± 0.053	247.51 ± 0.44	14.77 ± 0.03			
High	7.502 ± 0.001	247.89 ± 0.14	14.20 ± 0.03			
Extreme	7.205 ± 0.001	252.22 ± 0.15	14.25 ± 0.03			
**(b)**						
**Treatment**	**DIC** (**µmol/kg**)	**Alkalinity (µmol/kg)**	**pH**	**pCO**_**2**_ **(µatm)**	Ω _**aragonite**_	Ω _**calcite**_
Ambient	2100.1	2256.5	7.92	418.5	1.81	2.84
High	2227.0	2246.4	7.52	1175.5	0.77	1.20
Extreme	2291.6	2247.6	7.31	2012.9	0.49	0.76

The water was prepared by passing through a series of partially recirculating tanks. Oxygen and CO_2_ were stripped by bubbling nitrogen gas through membrane contactors^40^. The low-O_2_, low-CO_2_ water was the source water for 3 gas-controlled tanks where specified pH and water chemistry was maintained, continuously monitored^40^, and delivered at ~50 l*h^−1^ to flow-through plastic aquaria (56 × 30 × 25 cm) containing the algae vials.

One plastic flow-through aquaria was used for each pCO_2_ treatment, in a temperature controlled room with no outside light. Ten vials per algal taxon were assigned haphazardly to each of the three pCO_2_ levels along with 10 control vials (containing epoxy but no algae) and submerged vertically in the aquaria (top of vials under 15 cm of water) with vial tops open. Aquaria contained 4.2 L of water and water delivery was at 50 l*h^−1^, so water in aquaria and vials would be replaced every ~5 minutes. Vials were systematically mixed in the aquaria and rotated weekly. The vials were kept in the pCO_2_ treatments from March 7 to July 10, 2013. Interior and exterior walls of the vials, algal surfaces, and plastic aquaria were gently brushed weekly with a soft toothbrush to remove any accumulated diatoms which can reduce light levels and are normally removed by grazers. Algal surfaces were examined via digital photographs during the first month of the experiment, and under an Olympus dissecting microscope (40x magnification) immediately upon removal from MBARI, and there was no evidence of algal surface damage.

Sensors in the reservoir tank and each gas-controlled tank measured temperature, oxygen and carbon dioxide concentrations, and pH every 60 seconds (Table 1a). Oxygen and temperature were sensed using oxygen optodes (Aanderaa Inc., model 3835) immersed in the reservoir tank and in the three gas-controlled tanks, and pH was measured with Honeywell Durafet pH sensors. Temperature in the aquaria was maintained at a constant average of 14.4 °C (Table 1) because during upwelling events (when surface pH is naturally reduced) temperature remains low^38^. Two fluorescent bulbs ~0.5 m above each of the aquaria provided 13–15 µMol m^−2^ s^−1^ irradiance on a 12-hour cycle, measured at the experiment start and end using the LI-COR instrument. These levels mimicked average mid-day light levels at 9 m depth under kelp canopy at Hopkins Marine Station (measured in October of 2011 and March of 2012).

We also sampled water chemistry in the experimental aquaria three times monthly during the course of the experiment (March–July 2013) to measure salinity, total alkalinity, and dissolved inorganic content (DIC). On each collection date, nine water samples (three for each parameter/bin) were collected in 30 ml Borosilicate Glass serum bottles from beneath the surface of each bin. However, due to malfunction of water testing equipment for alkalinity and DIC, we were not able to measure the water samples until 4 months after the experiment terminated and data was unreliable. Therefore, in April 2014, we recreated the conditions of the experiment at MBARI using the same set points for the gas-controlled tanks, the same bins, and the same mass of coralline in each treatment bin. We let the system equilibrate for one week, then measured pH using a Shimadzu UV-1601 spectrophotometer, and re-sampled the water as described above (but with six replicates/parameter/pCO_2_ treatment) for salinity, total alkalinity, and DIC. For samples used to measure alkalinity and DIC, 10.9 μl HgCl_2_ was added to kill living organisms. Each bottle was sealed, stored in a dark refrigerator, and processed two days after collection. Salinity was measured with a YSI 3200 Conductivity instrument with a YSI 3252 cell, total alkalinity using a SI Analytics Titroline 6000 titrator, and DIC using a UIC Inc. Model 5015 CO2 Coulometer with a CM5230 Acidification module. We used the program CO2SYS to calculate pCO_2_ and Ω using the data from April 2014, with pH from the spectrophotometer readings and total alkalinity measured from collected water. The pCO_2_ values calculated were almost identical to the measures recorded within the gas-controlled tanks that fed water into experimental bins (Table 1b).

On July 10, vials were transported in cooled seawater to the Hopkins Marine Station for immediate use in larval settlement experiments. Lateral algal crust area was compared in before- and after-treatment photographs taken of individual vials (Canon Powershot S100 using Image J) as an indication of the effects of the pre-conditioning treatments. We do not report vertical growth because crusts were thin (≤0.2 mm), exhibited no visual change in vertical growth across treatments, and surface area is more important for maintenance of larval settlement space.

### Abalone settlement

Red abalone larvae from the Cayucos Abalone Farm (Cayucos, CA) were shipped overnight to the Hopkins Marine Station in seawater in a sterile Nalgene container surrounded by ice packs. Larvae were spawned on July 3, 2013, shipped on July 9^th^, and arrived on July 10, 2013. Because we were interested in how OA might alter the role of calcifying algae in species interactions, we exposed only algae crusts, not the larvae, to the different pCO_2_ treatments. To test whether OA affected algae cues for settling larvae, 95–110 red abalone larvae (*Haliotis rufescens*, 7-day old) were added to each of 7 haphazardly selected vials per taxon from each pCO_2_ treatment on July 10^th^ within hours of removal of algae from pCO_2_ treatments and immersion in ambient seawater (~14.2 °C and pH ~7.82). Abalone larvae were placed in sterile petri dishes and were inspected under an Olympus 40x dissecting microscope: all were actively swimming with no apparent abnormalities. Vials were filled with 65 mL of seawater (described above) leaving a 1 cm air space in each vial and 95–110 larvae were pipetted into each vial. Vials were closed with watertight polyethylene (LDPE) caps and put into a flowing seawater table to maintain temperature at 14 °C, under lights providing irradiance of ~15 µMol/m^2^/s on a 12 hour cycle. After 24-hours the number of settled (metamorphosed), swimming (veliger stage), and dead larvae were counted in each vial under a microscope. These counts were repeated 48 hours after abalone insertion.

### Statistical Analyses

All analyses were conducted in SYSTAT 13. Prior to conducting the pCO_2_ treatments, we assessed the surface area of CCRA assigned to the 3 treatments using analysis of variance (ANOVA). We used pCO_2_ treatments to pre-condition algae for later species interaction trials, which were multiple independent replicates. Data were normally distributed for algal area prior to treatment and no data transformation was applied.

We then tested for differences in growth to judge the effect of pre-conditioning. Because data were skewed, we used a logarithmic (base 10) transformation (plus the constant 20 to make all data positive as a few specimens had slight decline in healthy surface area). We compared the change in algal surface area between treatments and genera using a 2-way ANOVA with percent change in algal surface area (or cover) as the response variable, and pCO_2_ (3 levels) and algal genera (4 levels) as fixed, independent and orthogonal factors, and with the interaction term pH*genera. We then used a posthoc Tukey’s Honestly Significant Difference test to evaluate pairwise differences in growth between pCO_2_ levels and between algal genera.

To evaluate differences in abalone settlement across CCRA genera, we first ran an ANCOVA considering only the vials with CCRA (excluding non-CCRA control vials from each pCO_2_ treatment) so that we could include algal area as a predictor variable. We used the proportion of abalone that settled within 24 hours as the response variable and the predictor variables: algal pCO_2_ treatment (3 levels), algal genera (4 levels), algal surface area (as a covariate to account for any differences in the amount of algae in each vial), and the interaction between algal genera, pCO_2_ treatment, and algal area. We then ran an ANOVA with the same response variable and prior algal pCO_2_ treatment, substrate type (algal genera type or control vials with no algae), and the interaction between substrate type and prior algal pCO_2_ treatment. In both cases, the number of abalone settled was normally distributed and had homogeneous variances, so we did not use a transformation. Following the above analyses, where results were significantly different, we used a posthoc Ryan-Einot-Gabriel-Welsch (REGW-Q) test to evaluate which pCO_2_ or genera were significantly different from each other in inducing settlement. The datasets generated during and/or analysed during the current study are available from the corresponding author on reasonable request.

## Results

### Algal Species Identity

**Figure 1:**
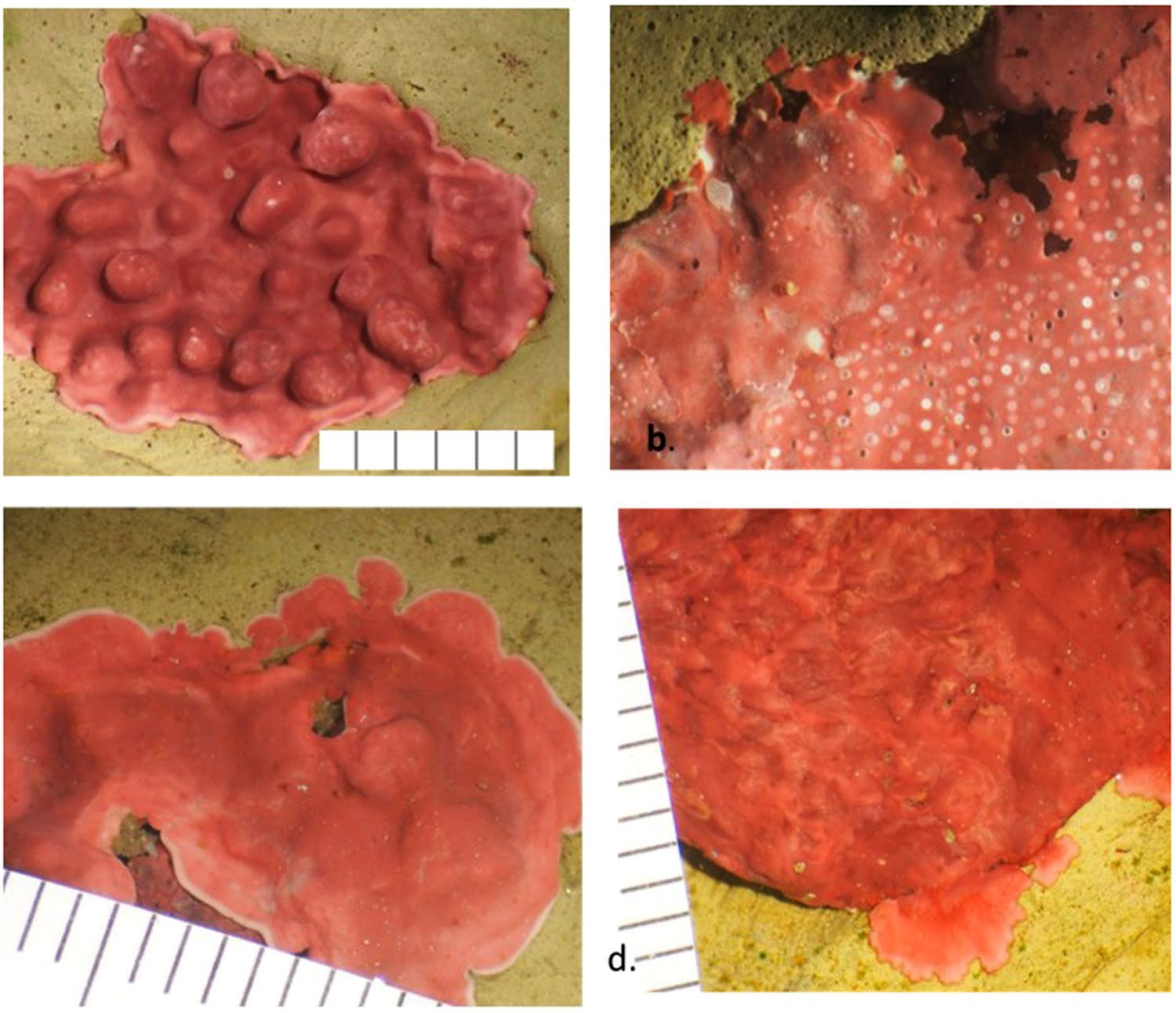
Surface morphology for each morphological group. Scientific names based on DNA sequencing of 5–10 samples/group. (**a**) Morphological group 1: Five samples of Group 1 were sequenced, with three identified as *Lithothamnion phymatodeum* and two as *L. glaciale*. *Lithothamnion* spp. specimens had raised multiporate conceptacles (~500 µm width) and short non-articulated protuberances that appear as distinct bumps on the surface (~1 × 1 × 1.5 mm). (**b**) Morphological group 2: Nine samples of this group sequenced as three related, undescribed species of *Lithophyllum*, some of which have also been found in northern Washington (PG pers obs). *Lithophyllum* spp. specimens had a very thin crust with numerous, small uniporate conceptacles (~150 µm width) that are flush with or slightly raised above the surface. (**c**) Morphological group 3: Six samples of this group were sequenced, with three identified as *Leptophytum adeyi* and three identified as a closely related, but undescribed *Leptophytum* species. *Leptophytum* spp. specimens were never found with conceptacles and had flowing growing margins, a very smooth surface, and fast growth in the laboratory compared to the other three groups. (**d**) Morphological group 4: Five samples were sequenced and all were a single lightly calcified aragonitic species of *Peyssonnelia*. These had a distinctive darker red coloration and flowing surface patterns. (Scale bars are in mm).

Four of five morphological groups of CCRA collected from cobbles in shallow kelp forests were consistently identified visually to genus (confirmed genetically), and these were used in the experiments. Three groups (each containing 2–3 closely related species) are common genera of CCA in the Northeast Pacific: *Lithothamnion spp*. (2 species), *Lithophyllum spp*. (3 species), and *Leptophytum spp*. (2 species; Fig. 1). The fourth group is a subtidal, lightly calcified aragonitic *Peyssonnelia* species (order Peyssonneliales) that may be undescribed (Fig. 1). These were the four most common genera on the cobbles collected from sites in the Monterey Bay. In addition, based on preliminary data from samples collected every 2.5 m on 30 m benthic transects in central California (13 transects from 2 sites in the Monterey Bay) and northern California (29 transects at 6 sites in Mendocino and Sonoma Counties), the 3 CCA genera (*Liththamnion, Lithophyllum*, and *Leptophytum*) are also common on reef substrates in kelp forests, accounting for 52% of the algal crust from transects in central California and 76% of crusts from transects in northern California. *Peyssonnelia* sp. was not found on any of the samples identified from transects and may be found only on cobbles rather than on rocky reefs (JKO, unpublished data).

### Algal Growth (Response to pre-conditioning period)

**Figure 2:**
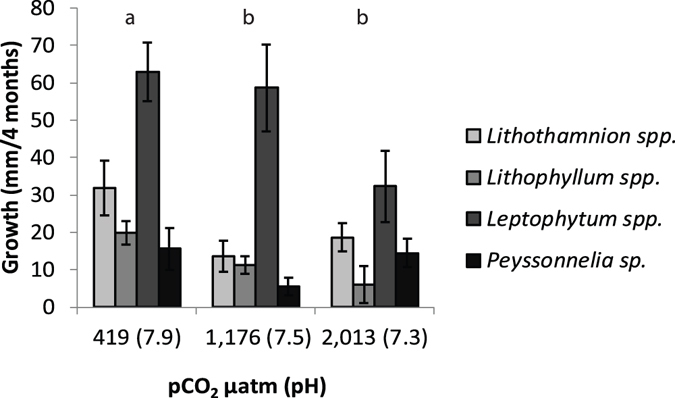
Change in surface area of the CCRA genera after four months in each of the pCO_2_ treatments, representing ambient conditions (419 µatm and pH of 7.9), high pCO_2_ (1,176 µatm and 7.5 pH), and extreme pCO_2_ (2,013 µatm and 7.3 pH). This data is shown to demonstrate that the pCO_2_ treatments had an effect on the CCRA. Letters above bars denote significant differences in growth considering all four genera.

Initial mean algal area (per vial) did not differ between pCO_2_ treatments (2-way ANOVA df = 2,6, F = 1.05, p = 0.35). The pre-conditioning of algae with elevated pCO_2_ negatively affected algal growth with significantly greater growth (+32.6 mm^2^) under normal pCO_2_ conditions compared to the high and extreme pCO_2_ conditions (+22.3 mm^2^ and + 17.8 mm^2^ respectively; Fig. 2, Tables 2a and 3a). Regardless of pCO_2_ treatment, there was variation in growth among the CCRA genera, with one genus, *Leptophytum* spp., growing significantly more than the others (Fig. 2, Tables 2a and 3b). All algal tissue appeared healthy at the end of the experiment with little bleaching (<7% of samples and with <2.6% of surface area affected).

**Table 2:** Statistical Results. (a) Effects of pre-condition pCO_2_ treatments on algal growth, (b) larval substrate preference (no control vials included) and accounting for algal area in vials, (c) larval settlement preference including control vials, and (d) larval substrate preference (no control vials included﻿).

Parameter	Statistical Test	Factor	Results
(a) Algal Growth	Two-way ANOVA	pCO_2_	df = 2,108, F = 7.93, p = 0.0006
		CCRA genera	df = 3,108, F = 20.59, p < 0.0001
		pCO_2_*CCRA genera	df = 6,108, F = 1.32, p = 0.25
(b) Larval Substrate Preference	ANCOVA	CCRA genera (no control)	df = 3,68, F = 7.60, p = 0.0002
		pCO_2_	df = 2,68, F = 1.27, p = 0.29
		CCRA surface area	df = 1,68, F = 0.44, p = 0.51
		pCO_2_*genera*area	df = 6,68, F = 0.60, p = 0.73
(c) Larval Substrate Preference (no control vials)	Two-way ANOVA	CCRA genera (& control)	df = 4,89, F = 50.91, p < 0.0001
		pCO_2_	df = 2,89, F = 1.68, p = 0.19
		pCO_2_*genera	df = 8,89, F = 0.56, p = 0.81

**Table 3:** Posthoc Tukey’s Honestly Signficant Difference (HSD) tests for algal growth following pCO_2_ treatments for the model in Table 2a with algal growth as the response variable and CCRA genera and pCO_2_ as predictor variables. Growth changes have been back transformed. (a) Growth differences by pCO_2_ (b) growth differences by CCRA genera.

(a)		
pCO_2_ Pairwise Comparison	Difference (mm)	p
Normal and High	10.29	0.01
High and Extreme	4.41	0.65
Normal and Extreme	14.70	0.008
**(b)**		
**Genera Pairwise Comparison**	**Difference** (**mm)**	**p**
*Leptophytum* spp. and *Lithothamnion* spp.	29.9	<0.0001
*Leptophytum* spp. and *Peyssonnelia* sp.	39.43	<0.0001
*Leptophytum* spp. and *Lithophyllum* spp.	39.9	<0.0001
*Lithothamnion* spp. and *Lithophyllum* spp.	9.0	<0.31
*Lithothamnion* spp. and *Peyssonnelia* sp.	9.5	=0.26
*Lithophyllum* spp. and *Peyssonnelia* sp.	0.57	=0.99

### Abalone Settlement

**Figure 3:**
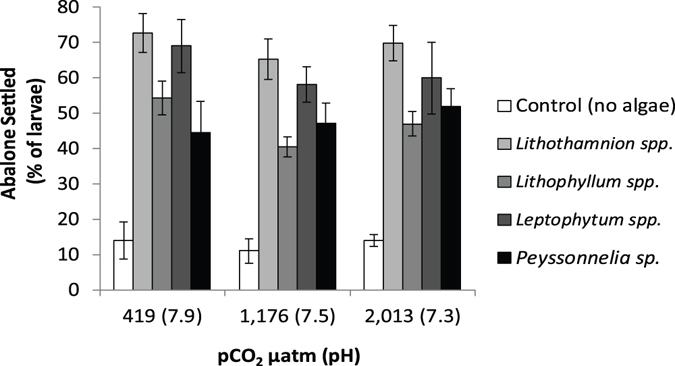
Abalone settlement (as percent of larval settled and attached) on the four CCRA genera and in control vials with no algae, and across the pCO_2_ treatments representing ambient conditions (419 µatm and pH of 7.9), high pCO_2_ (1,176 µatm and 7.5 pH), and extreme pCO_2_ (2,013 µatm and 7.3 pH).

The majority of abalone that settled (94%) did so within 24 hours, so we used the 24-hour numbers in analyses. We found no significant difference in the number of abalone settlers based on variation in CCRA surface area in vials, which ranged from 147 to 405 mm^2^ (Table 2b). Treatments that had no CCRA (control vials) had only 11% settlement compared to 48–69% in vials with CCRA (Table 2c and Fig. 3). There were significant differences in settlement rates between the four CCRA genera (p < 0.0001, Table 2c and Fig. 3): two genera, *Lithothamnion* spp. and *Leptophytum* spp., had higher settlement with 62 and 69% of larvae settling respectively while the other two genera, *Lithophyllum* spp. and *Peyssonnelia* sp, each had 48% of larvae settle (REGW-Q Test). To our knowledge, this is the first observation that *Peyssonnelia* may be an important settlement substrate for abalone. However, there was no change in settlement rates with prior pCO_2_ treatments of algae (the interaction term between algal genera and prior algal pCO_2_ treatment was not significant; Table 2c). Thus, while there was no significant difference in settlement associated with prior algal pCO_2_ treatment, there were strongly significant associations with presence or absence of CCRA as well as between algal genera.

## Discussion

One of the major unknowns in OA research is how species will respond to OA in a community context via species interactions^8–10^. For other environmental impacts like global warming, changes in species interactions have fundamental impacts on communities^41^. CCRA cover a high proportion of marine benthic substrates, are generally thought to be highly susceptible to OA^42^, and mediate multiple species interactions^43^. We tested the response of abalone larval settlement to CCRA that were preconditioned in OA treatments. Contrary to our hypothesis (that pre-conditioning CCRA with OA would disrupt larval settlement), we found that CCRA maintained their ability to induce larval abalone settlement despite prior exposure to strong OA (pCO_2_ of up to 2,013 µatm) for 4 months, at least when the algae but not the larvae were exposed.

### Response of CCRA to OA treatments

CCRA generally show diverse responses to experimentally increased pCO_2_ (Supplementary Table 1), likely reflecting the high diversity within this group. Prior OA experiments have found strong negative effects such as reduced growth and tissue integrity as well as increased likelihood of dissolution and tissue necrosis, but also sometimes no effect of increased pCO_2_ on growth^10^. While our ability to discuss the generality of changes in growth due to OA is constrained by the design of our experiment (e.g. one tank per algal pCO_2_ treatment), the level of CCRA growth reduction we observed is generally less severe than reported elsewhere. In our experiment, after 4 months, algal growth declined by 48% in the extreme pCO_2_ treatment (2,013 µatm). In four (of 6) prior laboratory and field studies in the extreme pCO_2_ range (1,400–2,200 µatm), coralline growth or cover was reduced by 93–100% (Supplementary Table 1). In our high pCO_2_ treatment (1,176 µatm), CCRA growth reduced by 32%, whereas in prior studies, cover or growth loss in similar pCO_2_ ranges varied from 22–100%, and most (9 of 13 studies) found >50% loss (Supplementary Table 1). Even in comparatively mild increased pCO_2_ treatments (600–850 µatm), prior studies have found cover or growth losses ranging from 15–100%, with 8 of 14 studies exhibiting >50% loss (Supplementary Table 1). Further, in our study, the fastest growing CCRA genus (*Leptophytum* spp.) maintained growth under high pCO_2_ and only reduced growth under the extreme pCO_2_ treatment (Fig. 2), indicating the potential for some taxa to resist at least moderate pH changes, which should be tested further.

### CCRA genus-specific settlement cues to abalone

Unlike corals, which have high species specificity for CCA^15, 19, 44^, red abalone larvae responded to cues from three genera of CCA and from the lightly calcified aragonitic crust, *Peyssonnelia* sp. To our knowledge, this is the first demonstration of *Peyssonnelia* providing abalone settlement cues. In a study on corals, *Peyssonnelia* was not found to be a particularly inductive substrate^45^. Although some CCRA genera in our study induced 1.4 times more settlement than others (Fig. 3), these differences pale when considering that there was >5 times more settlement when CCRA were present compared to when they were absent (10% compared to 48–69% settled). This finding is similar to the results of previous experiments where red abalone settled on multiple genera including the CCRA species *Lithothamnium californicum* and *L. glaciale*, *Lithophyllum* spp., *Clathromorphum circumscriptum*, and a CCRA non-coralline *Hildenbrandia dawsonii*, but did not settle when provided with foliose red, green, or brown algae^23^. There thus appears to be some functional redundancy among CCRA in providing settlement cues to red abalone. The functional redundancy in settlement cues found with red abalone is not necessarily the case for other abalone species (e.g. *Haliotis laevigata*), which show distinct settlement preferences among CCA species^46^.

### Abalone settlement on CCRA exposed to OA

We found that CCRA maintain settlement cues after prolonged and extreme OA exposure. This finding contrasts with previous studies on coral larvae showing settlement declines ranging between 20–86% on OA-treated CCRA^25, 26^, and showing settlement reductions at milder algal pCO_2_ treatments (600–1,300 µatm). The lack of OA-induced changes in larval settlement with elevated pCO_2_ in our experiment could be due to conditions not being extreme enough, short treatment times, or lack of power, but these scenarios are unlikely. Our OA treatments represented strong scenarios, with the most elevated treatment (2,013 µatm) set to higher pCO_2_ than global 2100 predictions^2, 36^. Further, pCO_2_ treatments had the expected negative effect on growth, with growth slowing significantly at high and extreme pCO_2_ treatments, especially for the fastest growing genus (*Leptophytum* spp.) in the extreme pCO_2_ treatment. The duration of the exposure of the algae to pCO_2_ treatments in this study was 126 days, well within the range of other similar laboratory studies (mean 138 days, range from 14–420 days; Supplementary Table 1), although one prior study found that CCRA showed a much stronger negative response at 420 days than at 90 days^47^. In California, upwelling of high pCO_2_ water lasts hours to days, not months, so our treatments were sufficient to evaluate potential effects of prolonged upwelling followed by the return of lower pCO_2_ water.

It should be noted that many impacts of OA on chemical communication are due to acid-base disturbances and subsequent impairment of neuronal ion channel function^48^ or due to changes in protonation of chemical signal compounds^49^ that lead to disrupted chemical signaling. Both types of effects would only be visible during direct and potentially prolonged exposure of both abalone larvae and CCRA to ocean acidification during settlement assays. However, our study focused on potential changes to CCRA provision of cues, rather than cue reception by larvae.

Our findings indicate that under expected future changes in pH in the California current system, cues to larval settlers like abalone that rely on CCRA may be retained following the cessation of upwelling and high pCO_2_. We found that several CCRA genera induced settlement (regardless of OA exposure history), suggesting functional redundancy that may provide an additional buffer against the effects of OA on loss of benthic coverage of any one coralline taxon. There is also evidence that the presence of algae, rather than percent cover, is sufficient for settlement^50^. In this experiment, there was no change in abalone settlement with algal surface area varying between 147 and 405 mm^2^. Direct contact by the larvae with the inducing algal surface is necessary for induction^23^, so at least locating CCRA is a requirement for abalone settlement, and this should be influenced by CCRA surface area. Nonetheless, reliance on presence rather than area will further buffer against the effects of slowed CCRA growth under future OA scenarios.

## Conclusions

Upwelling regions (like the California Current) have highly variable pCO_2_ conditions that can result in periodic exposure to pH well below normal^38, 39^. It has been suggested that for taxa that have evolved under conditions of pCO_2_ variation, OA effects may be less pronounced due to population acclimatization or adaptation^8^ than for species evolved under stable pCO_2_ conditions^51^. Thus, while California CCRA are susceptible to growth reductions under OA, they may be better able to withstand significant and prolonged changes in pCO_2_ than corallines from non-upwelling systems, and thus maintain chemical settlement cues. The hypothesis of environmental variability evolutionarily favoring physiological resistance to climate change is supported by studies showing that temperature variation is a key factor enhancing bleaching resistance in calcifying corals. This has been demonstrated in region-wide analyses^52^ and in field and laboratory experiments showing both acclimation and adaptation^53, 54^. Highly variable pCO_2_ may operate similarly, and background environmental variability may be a generally important factor in determining when to expect resistance. For example, in a fjord system with wide pCO_2_ fluctuations, barnacles showed a strong tolerance to high pCO_2_ (1,000 µatm)^55^. Though few species from the California upwelling system have been studied in an OA context^56^, prior studies have found evidence of OA adaptation in sea urchin (*S. purpuratus*) gametes^56^ and larvae^57^, and in mussel growth^56^. Abalone (*H. rufescens*) were found to have distinct differences in biomineralization genes between regions with different upwelling conditions, indicating local population adaptation^58^. Some calcifying species in California thus appear to tolerate broad pCO_2_ fluctuations or have sufficient genetic diversity to allow rapid evolution. For CCRA, individuals from a naturally variable tropical environment calcified 42% more than individuals from a uniform environment when experimentally placed under increased pCO_2_^59^. A study on a central California geniculate coralline, *Corallina vancouveriensis*, also found evidence of local adaptation, and it may be that the ability of CCRA spores to attach rapidly limits dispersal distance, restricts gene flow among populations, and increases the potential for local adaptation^60^.

Research is just revealing which taxa might be more vulnerable to OA and scaling up to species interactions and ecosystem functions is critical to predict and manage future changes. Species interactions of abalone larval settlers with CCRA in the California upwelling-dominated ecosystems appear to be resistant to the impacts of a four month history of OA exposure, and functional redundancy in settlement inducing CCRA may also buffer future OA impacts to abalone. The ability of CCRA to maintain chemical cues in this system may be due to periodic exposure to OA due to upwelling. If so, areas with variable OA environments like the California Current might represent areas with high resilience to OA in the future, compared with areas where pCO_2_ is more stable. Regardless, our findings demonstrate maintenance of some level of ecosystem function under OA in the California Current and possibly other upwelling ecosystems.

## Electronic supplementary material

Supplementary Table 1
