# Supplementary material for: Calcifying algae maintain settlement cues to larval abalone following algal exposure to extreme ocean acidification
Source: Sci Rep. 2017 Jul 18;7:5774. doi: 10.1038/s41598-017-05502-x (PMC5515930; doi:10.1038/s41598-017-05502-x)
Supplement: Supplementary file 1 — Supplementary Table 1 [file 41598_2017_5502_MOESM1_ESM.doc]

**Supplementary Materials**

­­Calcifying algae maintain settlement cues to larval abalone following algal exposure to extreme ocean acidification

Jennifer K. O’Leary^1,2^*, James P. Barry^3,Ŧ^, Paul W. Gabrielson^4,Ŧ^, Laura Rogers-Bennett^5,6, Ŧ^, Donald C. Potts^7, Ŧ^, Stephen R. Palumbi^1, Ŧ^ , Fiorenza Micheli^1, Ŧ^

^1^Hopkins Marine Station, Stanford University, Monterey, California, United States of America

^2^California Sea Grant, Department of Biology, California Polytechnic State University, San Luis Obispo. United States of America

^3^Monterey Bay Aquarium Research Institute, Moss Landing, California, United States of America

^4^Herbarium, University of North Carolina, Chapel Hill, North Carolina, United States of America

^5^Bodega Marine Laboratory, University of California, Davis, California, United States of America

^6^California Department of Fish and Wildlife, Marine Region, Bodega Bay, California, United States of America

^7^Department of Ecology and Evolutionary Biology, University of California, Santa Cruz, California United States of America

* Corresponding Author

Email: jkoleary@calpoly.edu (JO)

^Ŧ^ These authors contributed equally to this work

**Supplementary Table 1: Review of published studies investigating pCO2 effects on crustose coralline algae for response algal cover, algal growth, and invertebrate settlement.** Papers were found in ISIS Web of Knowledge using the search terms coralline (truncated), and crust (truncated) and “ocean acidification”, and the resulting 116 papers were scanned for the relevant response variables (growth, percent cover, or invertebrate settlement) and inclusion of crustose, non-geniculate corallines. This resulted in the 23 papers summarized here. Studies are grouped based on pCO_2_ range: mid (600-850 µatm), high (900-1300 µatm), and extreme (1400-2200 µatm). Data are expressed as proportional change (+/-) of treatment relative to ambient pCO_2_ (300-450 µatm pCO_2_). Where data is non-significant, we calculated a proportional change, but indicated n.s. after the number in the effect column. When studies covered multiple seasons, we averaged results. When studies included a second factor (e.g. temperature or light), we only report independent effects of pCO_2_ (we report results for ambient temperature and light conditions). Where dissolution occurred in growth studies, we did not show loss via dissolution but simply indicate complete stop of growth (-1.0). Where data was graphed, we extracted values using WebPlotDigitizer . *Studies where larvae were also exposed to pCO_2_ treatments and effect on larvae versus algae is unknown.

| **Region** | **Response Variable** | **IPCC Scenario** | **Length**  **Exposed**  **(days)** | **Effect** | **Morphology** | **Citations** |
| --- | --- | --- | --- | --- | --- | --- |
| Antarctica | Cover | High | 47 | -0.55 n.s. | crust | Schoenrock et al 2015^1^ |
| Temperate  (Pacific NE) | Growth | Mid | 27 | -1.0 n.s. | crust | Kram et al 2016^2^ |
| Temperate  (Mediterranean) | Cover | Mid | *in situ* | -1.0 | crust | Porzio et al 2011^3^ |
|  |  | High | *in situ* | -0.71 | crust, (geniculate not separated) | Hall Spencer et al 2008^4^ |
|  |  |  | 14 | -0.60 | crust | Hall Spencer et al 2008^4^ |
|  |  |  | 420 | -0.34 | crust | Kamenos et al 2016^5^ |
|  |  |  | *in situ* | -1.0 | crust | Linares et al 2015^6^ |
|  |  | Extreme | 14 | -0.93 | crust | Hall Spencer et al 2008^4^ |
|  |  |  | 420 | 0.58 | crust | Kamenos et al 2016^5^ |
|  |  |  | *in situ* | -1.0 | crust | Martin et al 2008^7^ |
|  |  | Extreme | *in situ* | -1.0 | crust | Porzio et al 2011^3^ |
| Temperate  (North Sea) | Growth | Mid | 90 | -0.22 | crust | Ragazzola et al 2013^8^ |
|  |  |  | 300 | -0.64 | crust | Ragazzola et al 2013^8^ |
|  |  | High | 90 | -0.33 | crust | Ragazzola et al 2013^8^ |
|  |  |  | 300 | -0.61 | crust | Ragazzola et al 2013^8^ |
| Tropical | Cover | Mid | 180 | -0.43 | crust | Vogel et al 2016^10^ |
|  |  |  | 420 | -0.82 | crust | Crook et al 2016^11^ |
|  |  |  | 60 | -0.39 | crust | Doropoulos et al 2012^12^ |
|  |  |  | in situ | -0.67 | crust | Fabricius et al 2015^13^ |
|  |  |  | 390 | -0.63 | crust | Fabricius et al 2015^13^ |
|  |  |  | 56 | -0.91 | crust | Kuffner et al 2007^14^ |
|  |  |  | 300 | -0.86 | crust | Jokiel et al 2008^15^ |
|  |  |  | 75 | -0.38 | crust | Ordoñez et al 2016^16^ |
|  |  | High | 60 | -0.28 | crust | Doropoulos et al 2012^12^ |
|  |  |  | 35 | -0.84 | crust | Dutra et al 2016^17^ |
|  |  |  | 75 | -0.79 | crust | Ordoñez et al 2014^16^ |
|  |  |  | 180 | -1.0 | crust | Vogel et al 2016^10^ |
|  |  |  | 42 | -0.64 | crust | James et al 2014^18^ |
|  | Growth | Mid | 27 | -0.16 | crust | Agegian 1985^19^ |
|  |  | High | 14 | -1.0 | crust | Johnson et al 2014^20^ |
|  |  | Extreme | 14 | -1.0 | crust | Johnson et al 2014^20^ |
|  | Invert Recruitment | Mid | 3 | -0.47  (-0.34 to -0.57) | crust | Doropoulos & Diaz-Pulido 2013*^21^ |
|  |  |  | 60 | -0.86 | crust | Doropoulos et al 2012^12^ |
|  |  |  | 42 | -0.20 | crust | Webster et al 2013^22^ |
|  |  | High | 3 | -0.62  (-0.47 to -0.86) | crust  crust | Doropoulos & Diaz-Pulido 2013*^21^ |
|  |  |  | 60 | -0.50 ns | crust | Doropoulos et al 2012^12^ |
|  |  |  | 42 | -0.23 | crust | Webster et al 2013^22^ |
|  |  | Extreme | 42 | -0.52 | crust | Webster et al 2013^22^ |
|  | Invert Selectivity | Mid | 60 | -1.0 | crust | Doropoulos et al 2012^12^ |
|  |  | High | 60 | -1.0 | crust | Doropoulos et al 2012^12^ |

**Supplementary Literature Cited**

1. Schoenrock, K. M. et al. Climate change confers a potential advantage to fleshy Antarctic crustose macroalgae over calcified species. *J. Exp. Mar. Bio. Ecol.* **474**, 58–66 (2016).

2. Kram, S. et al. Variable responses of temperate calcified and fleshy macroalgae to elevated pCO2 and warming. *Ices J. Mar. Sci*. **73**, 250–262 (2016).

3. Porzio, L., Buia, M. C. & Hall-Spencer, J. M. Effects of ocean acidification on macroalgal communities. *J. Exp. Mar. Bio. Ecol.* **400**, 278–287 (2011).

4. Hall-Spencer, J. M. et al. Volcanic carbon dioxide vents show ecosystem effects of ocean acidification. *Nature* **454**, 96–9 (2008).

5. Kamenos, N., Perna, G., Gambi, M., Micheli, F. & Kroeker, K. Coralline algae in a naturally acidified ecosystem persist by maintaining control of skeletal mineralogy and size. *Proc. R. Soc. B* **238**: 20161159(2016).

6. Linares, C. et al. Persistent natural acidification drives major distribution shifts in marine benthic ecosystems. *Proc. R. Soc. B Biol. Sci.* **282**, 20150587 (2015).

7. Martin, S. et al. Effects of naturally acidified seawater on seagrass calcareous epibionts. *Biol. Lett.* **4**, 689–92 (2008).

8. Ragazzola, F. et al. Phenotypic plasticity of coralline algae in a High CO_2_ world. Ecol. Evol. 3, 3436–46 (2013).

9. Anthony, K. R. N., Kline, D. I., Diaz-Pulido, G., Dove, S. & Hoegh-Guldberg, O. Ocean acidification causes bleaching and productivity loss in coral reef builders. *Proc. Natl. Acad. Sci. U. S. A*. **105**, 17442–6 (2008).

10. Vogel, N. et al. Interactive effects of ocean acidification and warming on coral reef associated epilithic algal communities under past, present-day and future ocean conditions. *Coral Reefs* **35**, 715–728 (2016).

11. Crook, E. D. et al. Recruitment and succession in a tropical benthic community in response to in-situ ocean acidification. *PLoS One* **11**, 1–16 (2016).

12. Doropoulos, C., Ward, S., Diaz-Pulido, G., Hoegh-Guldberg, O. & Mumby, P. J. Ocean acidification reduces coral recruitment by disrupting intimate larval-algal settlement interactions. *Ecol. Lett.* **15:** 338–346 (2012).

13. Fabricius, K. E., Kluibenschedl, A., Harrington, L., Noonan, S. & De’ath, G. In situ changes of tropical crustose coralline algae along carbon dioxide gradients. *Sci. Rep.* **5**, 9537 (2015).

14. Kuffner, I. B., Andersson, A. J., Jokiel, P. L., Rodgers, K. S. & Mackenzie, F. T. Decreased abundance of crustose coralline algae due to ocean acidification. *Nat. Geosci.* **1**, 114–117 (2007).

15. Jokiel, P. L. et al. Ocean acidification and calcifying reef organisms: a mesocosm investigation. *Coral Reefs* **27**, 473–483 (2008).

16. Ordoñez, A., Doropoulos, C. & Diaz-Pulido, G. Effects of ocean acidification on population dynamics and community structure of crustose coralline algae . *Biol. Bull.* **226**, 255–268 (2014).

17. Dutra, E., Koch, M., Peach, K. & Manfrino, C. Tropical crustose coralline algae individual and community responses to elevated pCO_2_ under high and low irradiance. *Ices J. Mar. Sci.* **73**, 803–813 (2016).

18. James, R. K., Hepburn, C. D., Cornwall, C. E., McGraw, C. M. & Hurd, C. L. Growth response of an early successional assemblage of coralline algae and benthic diatoms to ocean acidification. *Mar. Biol.* **161**, 1687–1696 (2014).

19. Agegian, C. The biochemical ecology of *Porolithon gardineri* (Foslie). (University of Hawaii, 1985).

20. Johnson, M. D., Moriarty, V. W. & Carpenter, R. C. Acclimatization of the crustose coralline alga Porolithon onkodes to variable pCO₂. *PLoS One* **9**, e87678 (2014).

21. Doropoulos, C. & Diaz-Pulido, G. High CO_2_ reduces the settlement of a spawning coral on three common species of crustose coralline algae. *Mar. Ecol. Prog. Ser.* **475**, 93–99 (2013).

22. Webster, N. S., Uthicke, S., Botté, E. S., Flores, F. & Negri, A. P. Ocean acidification reduces induction of coral settlement by crustose coralline algae. *Glob. Chang. Biol.* **19**, 303–315 (2013).
